# Exoskeleton-based training improves walking independence in incomplete spinal cord injury patients: results from a randomized controlled trial

**DOI:** 10.1186/s12984-023-01158-z

**Published:** 2023-03-24

**Authors:** Ángel Gil-Agudo, Álvaro Megía-García, José Luis Pons, Isabel Sinovas-Alonso, Natalia Comino-Suárez, Vicente Lozano-Berrio, Antonio J. del-Ama

**Affiliations:** 1 Biomechanics and Technical Aids Department, National Hospital for Paraplegics, SESCAM, Finca la Peraleda s/n, 45071 Toledo, Spain; 2 Physical Medicine and Rehabilitation Department, National Hospital for Paraplegics, SESCAM, Toledo, Spain; 3 Neurorehabilitation and Biomechanics Unit (HNP-SESCAM), Associate Unit CSIC, Toledo, Spain; 4 Toledo Physiotherapy Research Group (GIFTO), Faculty of Physiotherapy and Nursing, Castilla La Mancha University, Toledo, Spain; 5 Legs and Walking Lab, Shirley Ryan Ability Laboratory (Formerly Rehabilitation Institute of Chicago), Chicago, IL USA; 6 Department of Physical Medicine and Rehabilitation, Feinberg School of Medicine, Northwestern University, Chicago, IL USA; 7 Department of Biomedical Engineering, McCormick School of Engineering and Applied Science, Northwestern University, Chicago, IL USA; 8 Department of Mechanical Engineering, McCormick School of Engineering and Applied Science, Northwestern University, Chicago, IL USA; 9 Neural Rehabilitation Group, Cajal Institute, Spanish National Research Council (CSIC), Madrid, Spain; 10 Rey Juan Carlos University, Electronic Technology Area, Móstoles, Spain

**Keywords:** Neurological rehabilitation, Spinal cord injury, Robotic exoskeleton, Walking

## Abstract

**Background:**

In recent years, ambulatory lower limb exoskeletons are being gradually introduced into the clinical practice to complement walking rehabilitation programs. However, the clinical evidence of the outcomes attained with these devices is still limited and nonconclusive. Furthermore, the user-to-robot adaptation mechanisms responsible for functional improvement are still not adequately unveiled. This study aimed to (1) assess the safety and feasibility of using the HANK exoskeleton for walking rehabilitation, and (2) investigate the effects on walking function after a training program with it.

**Methods:**

A randomized controlled trial was conducted including a cohort of 23 patients with less than 1 year since injury, neurological level of injury (C2-L4) and severity (American Spinal Cord Injury Association Impairment Scale [AIS] C or D). The intervention was comprised of 15 one-hour gait training sessions with lower limb exoskeleton HANK. Safety was assessed through monitoring of adverse events, and pain and fatigue through a Visual Analogue Scale. LEMS, WISCI-II, and SCIM-III scales were assessed, along with the 10MWT, 6MWT, and the TUG walking tests (see text for acronyms).

**Results:**

No major adverse events were reported. Participants in the intervention group (IG) reported 1.8 cm (SD 1.0) for pain and 3.8 (SD 1.7) for fatigue using the VAS. Statistically significant differences were observed for the WISCI-II for both the “group” factor (F = 16.75, p < 0.001) and “group-time” interactions (F = 8.87; p < 0.01). A post-hoc analysis revealed a statistically significant increase of 3.54 points (SD 2.65, p < 0.0001) after intervention for the IG but not in the CG (0.7 points, SD 1.49, p = 0.285). No statistical differences were observed between groups for the remaining variables.

**Conclusions:**

The use of HANK exoskeleton in clinical settings is safe and well-tolerated by the patients. Patients receiving treatment with the exoskeleton improved their walking independence as measured by the WISCI-II after the treatment.

## Background

One of the most limiting consequences of spinal cord injury (SCI) is the complete or partial paralysis of the lower limbs. Still, independent from the severity of the spinal injury, the time after lesion, or age at the time of injury, the restoration of walking is given high priority by subjects with SCI [1, 2]. Therapies for rehabilitation of walking ability after SCI aim at exploiting neural plasticity while providing strength training of the remaining active muscles and optimizing functional compensating strategies [3]. Intensive stepping training can activate the neural circuits responsible for the generation of rhythmic patterns of movement or CPG [17]. This activation can induce plastic changes both at the level of the spinal cord and in the motor-sensory cortex in people with incomplete spinal cord injury, if the activation, thus the stepping training, is maintained sufficiently over time [4–6]. Furthermore, repetitive and task-specific training promotes simultaneous activation of sensory and motor pathways that can select and reinforce spinal circuits, thus improving the ability to perform the practiced movement successfully [7, 8].

Functional walking rehabilitation has therefore to be intensive and task-oriented. Robotic devices were developed more than 20 years ago to allow for intensive, weight-bearing, stepping training with precise movement control [9, 10]. However, there is still no solid evidence about the superiority of robotic rehabilitation over conventional therapy [11–16]. The training environment provided through robotic trainers alter, or even remove, important factors for rehabilitation -e.g. full limb loading during stance, visual and vestibular inputs amongst others-. This non-realistic training environment has been listed as one of the several factors that might explain why walking outcomes are not proportional to the intensity of treatment [17].

In this sense, ambulatory robotic exoskeletons have been proposed in the last decade to provide task-specific, ambulatory overground walking training, mainly in incomplete SCI patients that have improvement prognosis. In contrast to robotic trainers, robotic exoskeletons may optimally challenge the patient in the domains of balance and physical exercise, while providing walking-consistent visual and functional feedback. The safety and comfort of robotic exoskeletons as a rehabilitation tool have been previously assessed in patients with acute and chronic SCI [18–23], enabling safe walking while reducing energy expenditure compared to passive bracing in patients with thoracic injuries [15, 24]. Still, the robustness and technological maturity of these devices is improving continuously, although the protocols and clinical indications for their use have not evolved so quickly.

While some studies have reported improvements in some functional and spatial–temporal variables of walking (i.e. gait speed), the literature concerning the walking outcomes due after a rehabilitation program with robotic exoskeletons is nowadays limited and nonconclusive. One of the main rationales is related to the differences in interventions (robot type and control, treatment time, and the number of sessions), which makes it difficult to obtain a clinically reliable conclusion [25]: most of the reported studies have been conducted on small samples and with quite heterogeneous protocols [26]. To date, the study carried out on the largest patient sample with SCI is that on exoskeletons manufactured by Ekso Bionics [24, 27], in which gait training was assessed in different sub-groups of a heterogeneous SCI population.

Currently, one of the main issues to be addressed is whether walking training with robotic exoskeletons generates improvements in the person’s walking ability, and what device-related rationales are responsible for the outcomes. In this article, we present the results of a prospective, randomized, and comparative study conducted to assess the clinical efficacy of a novel robotic lower-limb robotic exoskeleton named HANK. While the main features of this exoskeleton are similar to others available on the market or tested in similar studies, there are slight differences that justify the present study. We also provide evidence on the impact of delivering walking training with this kind of technology in incomplete SCI patients, contributing to answering the fundamental question related to the outcomes of robotic walking rehabilitation conveyed with exoskeletons [28, 29]. Therefore our objectives were: (1) to assess the safety and adherence to the robotic-assisted walking training as provided with the HANK exoskeleton by individuals with incomplete SCI, and (2) to assess the changes in walking function after a training program with HANK exoskeleton compared to traditional walking therapy in patients with non chronical condition. The hypothesis was that the treatment with the exoskeleton would provide better functional walking outcomes than traditional overground gait therapy.

## Methods

**Fig. 1:**
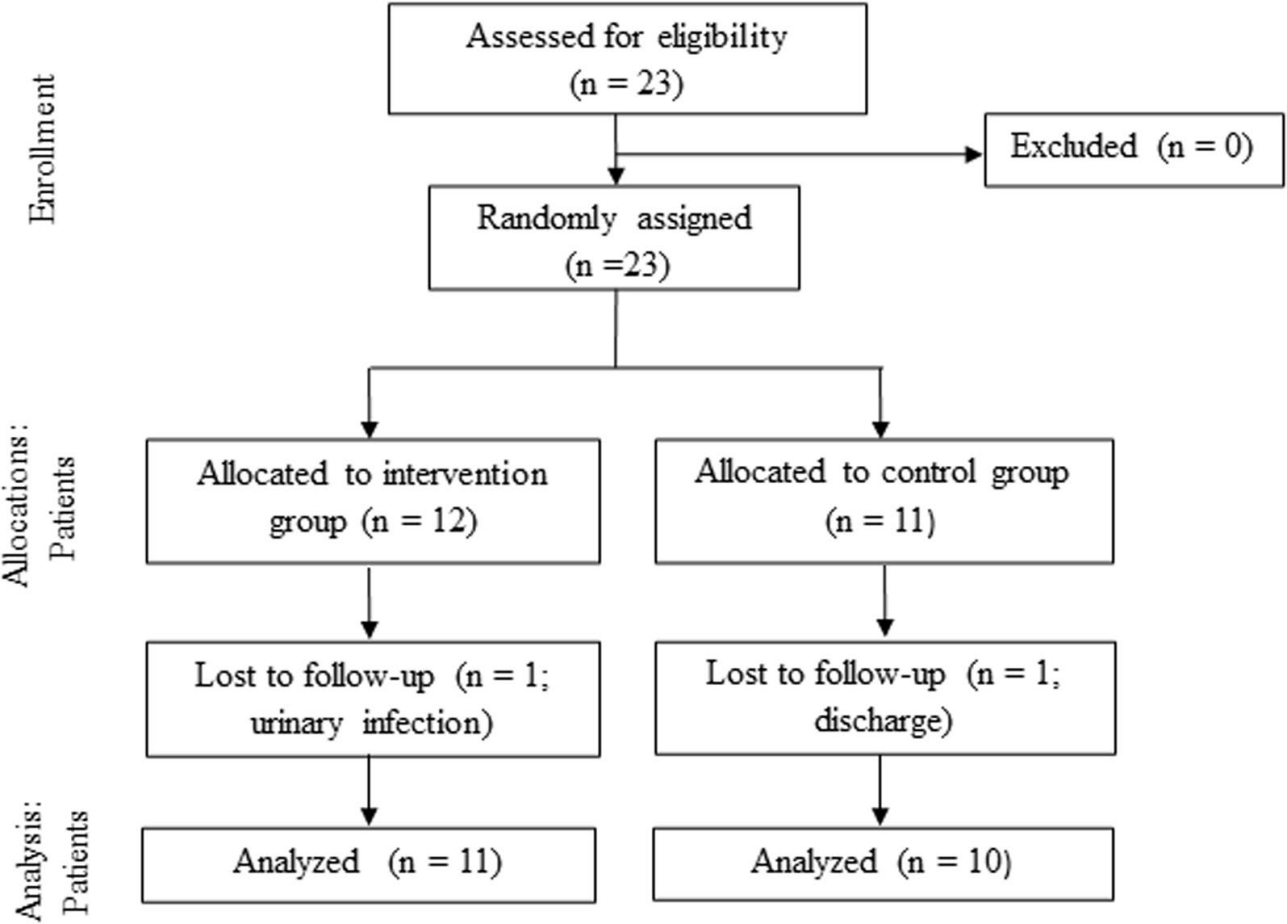
CONSORT flow diagram

A prospective, randomized, comparative study was carried out (Fig. 1) in which all the data was collected and analyzed following CONSORT (Consolidated Standards of Reporting Trials) guidelines for randomized trials of nonpharmacological treatments [30]. The evaluator was blinded and the participants were recruited via non-probabilistic convenience sampling and were randomly distributed into the two study groups: exoskeleton gait training (intervention group, IG) or traditional gait training (control group, CG). Randomization was performed using Randomizer.org. The present study was approved by the local Ethical Committee in Hospital Virgen de la Salud of Toledo, Spain (Ref. No. 39; 07/02/2007) and conforms to the Helsinki Declaration of 1975, as revised in 2020. The clinical trial was registered at ClinicalTrials.gov (NCT03477123). Initially, this study was registered for Exo-H2 but finally, we chose to perform the study using an improved version of the device named “HANK”. As explained below, the improvements only related to the mechanical design of the gearboxes and the device’s electronics, remaining the mechanical structure and control unchanged. Therefore, from the treatment perspective, both Exo-H2 and HANK exoskeletons are completely equivalent.

### HANK exoskeleton

**Fig. 2:**
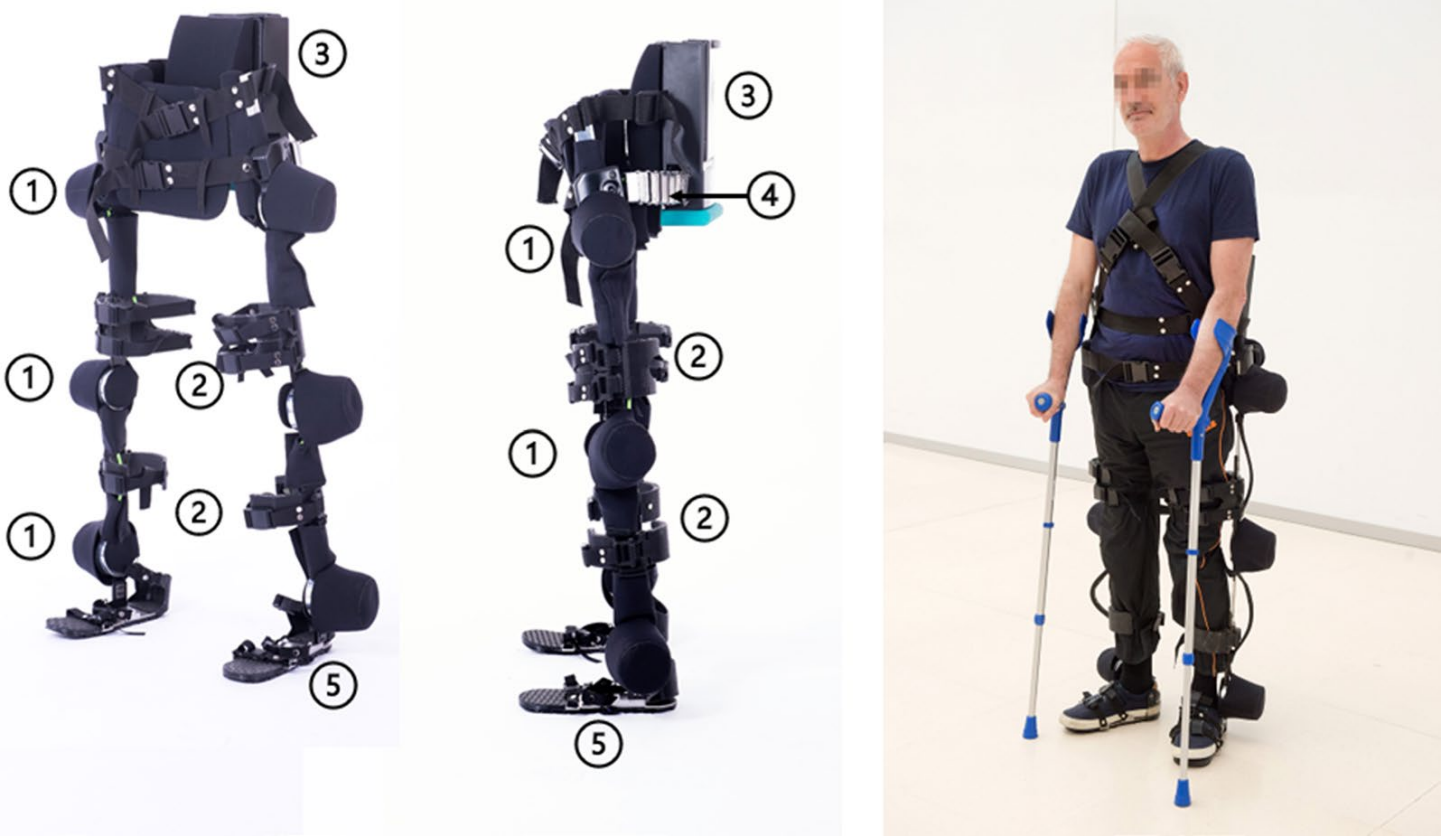
HANK exoskeleton; **a** oblique view, **b** lateral view, **c** HANK exoskeleton on a patient carrying two crutches; (1) actuators, (2) attachment and fitting, (3) backpack tat containing battery, main microprocessor and the communication electronics connects, (4) flexible arm for adaptation to pelvis, (5) force-sensing-resistors for measuring foot-floor contact

The HANK exoskeleton (Gogoa Mobility Robots SA, Spain) is an updated version of Exo-H2 exoskeleton, previously described and studied in patients with stroke [31] and incomplete SCI [32]. Modifications were introduced to Exo-H2 that led to HANK, involving some improvements. Indeed, HANK’s ankle, knee and hip actuators are now more compact (see 1 in Fig. 2), and the physical interface features 3D-printed rigid cuffs with padding for the leg and thigh that are tightened with straps and magnetic locks (see 2 in Fig. 2). Left and right legs are connected to a rigid backpack (see 3 in Fig. 2) via a flexible structure that allows it to bend in the coronal planes, hence it can be adapted to different pelvis widths while retaining sufficient stiffness in the sagittal and frontal planes (see 4 in Fig. 2). The backpack contains the battery, the main microprocessor and the communications electronics. The control interface is based on software implemented on an Android tablet, which connects via Bluetooth to the main controller. The guidance force can be set as a percentage, ranging from 100% as completely rigid position control to 0% with free movement. While this might allow setting the optimum assistance to the specific patient capacity to, hypothetically, provide the best outcome [33], there are no clinical guidelines stating the criteria to set and modulate guidance force related to either patient capacity or progression during therapy. Therefore, and in line with several studies, we set the guidance to 100% providing rigid trajectory guidance.

### Participants

This clinical study focused on individuals with incomplete SCI less than 1 year since injuy. The cohort was recruited at the National Hospital for Paraplegics (Toledo, Spain) and the eligibility criteria were: age between 16 and 70 years old; C2-L4 level of injury; severity (American Spinal Cord Injury Association Impairment Scale [AIS] C or D); time since injury less than 12 months; enough strength in the upper limbs necessary to handle a walker or crutches (triceps brachial muscle balance ≥ 3); the capacity to tolerate standing; spasticity in lower limb muscles ≤ 2 according to the Modified Ashworth scale; and signing of informed consent. Participants were excluded if they were pregnant or had: any other neurological condition apart from SCI; a recent lower extremity fracture (less than 1 year); irreducible contracture or arthrodesis in the lower limb joints; ulcers sores at the contact points with the exoskeleton. Although to facilitate recruitment, the age range of the inclusion criteria was very wide, most of the subjects were between 33 and 59 years old except 4 who were 19, 23, 64 and 71 bearing the heterogeneity of the cohort.

### Intervention procedures

The IG training protocol consisted of 15 robotic ambulatory gait training sessions, each session lasting 30 min. Three sessions were performed on non-consecutive days each week for 5 consecutive weeks. The total duration of each session was about 1 h, which included 20 min to put on and remove the exoskeleton, 30 min for the walking training, 5 min of rest and 5 min to register the variables assessed. The training was conducted indoors on a smooth level surface in the physiotherapy room. External support was always used depending on the participant’s functional status, preferences, and ability: parallel bars, walker or two crutches. Besides, a researcher -physiotherapist- provide contact guarding, and another -engineer- was in charge of the exoskeleton control. Feedback was provided to patients of the IG during the sessions, were instructed on maintaining their walking pace. After the sessions, we provided feedback regarding the distance walked. The CG rehabilitation program was comprised of 15 sessions, 30 min. long, of a traditional gait training program (analytical mobilization, strengthening exercises for the lower limbs and gait re-education when possible using parallels), distributed similarly as in the IG: 3 sessions each week for 5 weeks. Neither the participants in the IG group nor those in the CG group modified their usual medications or the rehabilitation programs during the study. In other words, both the IG and the CG groups received additional walking treatment due to their participation in the study.

### Outcome variables

Safety was closely monitored during the study. It was assessed by registering and analyzing any adverse event, particularly those related to skin lesions, falls and pain. The participants’ skin was inspected before and after each session based on the US National Pressure Ulcer Advisory Panel and European Pressure Ulcer Advisory Panel pressure ulcer classification system [34]. After each training session, a Visual Analogue Scale (VAS, 0–10 cm) was used to record the pain and fatigue perceived during the session [35]. Feasibility and adherence to the treatment were analyzed by recording the number of participants who completed the treatment, along with the pain and fatigue.

Clinical and functional data were measured at baseline and end of the training period (post-intervention), without the exoskeleton by another physiotherapist than those who conducted the treatment. The severity and level of SCI were determined through neurological examination according to the International Standards for Neurological Classification of Spinal Cord Injury (ISNCSCI) [36]. The Lower Extremity Motor Score (LEMS) [37] was used as an outcome measure rather than the total upper and lower extremity motor ability [38, 39]. The performance of all participants in the following tests was evaluated: 10 Meter Walking Test (10MWT) [40], Timed Up and Go (TUG) [41], 6 Minute Walking Test (6MWT) [40], Walking Index for SCI (2nd version: WISCI-II) [42], and Spinal Cord Independence Measure-III (3 version: SCIM-III) [43].

### Statistical analysis

Statistical analyses were performed using the commercial IMB SPSS v22 software package (IBM Corporation, Armonk, NY).The sample size was calculated based on the LEMS score as the main variable, considering a mean difference of 8.2 and a standard deviation of 10 that was obtained from a pilot study [32]. To achieve a Type I error (α) of 0.05 and a power of 80% a sample size of 24 subjects in each group is needed. The homogeneity between the groups in terms of age, gender, time since injury, WISCI-II and LEMS was evaluated with a Levene´s test for equality of variances. A two-way repeated-measures ANOVA, with the “time” factor (baseline and post-intervention) and the “group” factor (IG and CG groups), was performed to compare differences in the motor score (LEMS) and the functional gait variables (10MWT, TUG, 6MWT, WISCI-II and SCIM-III). The Greenhouse–Geisser correction was applied to correct for non-sphericity, and a Bonferroni post-hoc multiple comparison test was used to highlight specific differences between evaluation time and groups. Participants who did not complete the study were not included in the statistical analysis. A p-value ≤ 0.05 was considered statistically significant.

## Results

The eligibility criteria were met by 23 participants with incomplete SCI within the recruitment period: 12 out of the 23 were randomly allocated to the IG and 11 of the 23 to the CG. We experienced a slow recruitment rate due to different but common reasons [44] and reached the time limit stated by our financial sponsorship, and therefore had to stop the study without reaching the number of participants (48, 24 per group). Besides, we had 2 drop-outs due to rationales not related to the study: one had urine infection and another was discharged before finalizing the study. Therefore, 21 participants completed the study protocol and were considered for analysis. The demographic and clinical characteristics of the participants are shown in Table 1. The majority of our patients were less than 6 months post-injury. Both groups were homogeneous for age, gender, time since injury, injury level, AIS scale, LEMS and WISCI-II (Table 1).

**Table 1:** Patients´ baseline characteristics

Characteristic	Intervention group (IG)	Control group (CG)	Levene’s test value (p-value)
Age	41 (12.39)	51.8 (11.93)	F = 0.11 (p = 0.74)
Sex			
Male	7	8	F = 0.12 (p = 2.65)
Female	4	2	
Injury level			
C2–C8	0	1	F = 0.55 (p = 0.47)
T1–T6	4	1	
T7–L1	4	5	
L2–L4	3	3	
AIS			
C	8	4	F = 1.26 (p = 0.27)
D	3	6	
Time since injury (months)	4.82 (1.3)	5.55 (2.3)	F = 2.17 (p = 0.16)
LEMS (0–50)	27.82 (11.09)	33.3 (7.63)	F = 0.9 (p = 0.36)
WISCI-II (0–20)	11.91 (4.25)	11.7 (3.8)	F = 0.03 (p = 0.87)

Variables are expressed as mean (SD). (n), number of participants able to finish the study

AIS: American Spinal Injury Association Impairment Scale; LEMS: Lower Extremity Motor Scale; WISCI-II: Walking Index for Spinal Cord Injury II. *p ≥ 0.05

### Safety, feasibility, pain and fatigue

No participants experienced a fall during the study, yet two IG participants suffered mild skin erythema in the tibia contact zone during the first session that was related to the strap. This issue was resolved by adding padding to the specific zones and the lesions disappeared within one day. In addition, mild neck and shoulder muscle pain was reported 24 h after the training session in 6 IG patients, which was probably related to the use of walking aids. Concerning the pain and fatigue perceived after each IG session, the participants rated the pain as 1.8 (SD 1.0) cm and the fatigue as 3.8 (SD1.7) cm in a VAS (0–10 cm).

### Effects on LEMS

The two-way ANOVA showed significant differences for the “time” factor in LEMS (F = 19.9, p < 0.001) but not for the “group” factor (F = 0.16, p = 0.69) and for the “time-group” interaction (F = 0.5, p = 0.4). A post-hoc pairwise comparison with Bonferroni correction showed a significant increase in the LEMS score after the intervention of 4.4 (SD 5.4) points in the IG (p = 0.003, η^2^ = 0.45) and of 3 (SD 2.7) points in the CG (p = 0.04, η^2^ = 0.45: see Table 2), although no differences were found between the groups (p = 0.27, η^2^ = 0.03).

**Table 2:** Lower extermity motor score (LEMS) assessment

	Intervention Mean (SD)			Pairwise comparison (P-value, η^2^)	
	Baseline	Post-intervention	Change	Baseline vs. Post-intervention	IG vs. CG
IG	27.82 (11.09)	32.27 (9.3)	4.45 (5.37)	0.00*, η^2^ = 0.45	0.268, η^2^ = 0.03
CG	33.3 (7.63)	36.3 (7.24)	3 (2.66)	0.04*, η^2^ = 0.45	

CG: control group; IG: intervention group; *significant differences (p < 0.05). η^2^: calculated effect size

### Effects on the functional scales

The functional outcomes at baseline and post-intervention were assessed (Table 3 including effect size), and a two-way ANOVA showed significant differences for the “time” factor in the 10MWT (F = 15.54, p < 0.001, TUG (F = 36.37, p < 0.0001) and 6MWT (F = 17.0, p < 0.001), yet no significant differences were found for the “group” factor (10MWT: F = 0.16, p = 0.69; TUG: F = 0.0, p = 0.99; 6MWT: F = 0.1, p = 0.76) and for the “time-group” interaction (10MWT: F = 0.72, p = 0.4; TUG: F = 3.5, p = 0.08; 6MWT: F = 0.6, p = 0.45). The walking speed increased 0.2 m/s (SD 0.2) in the IG (p < 0.05) and 0.1 (SD0.17) m/s CG (p < 0.05), while the TUG improved significantly after intervention in both groups. The time necessary to carry out the test decreased 13.2 (SD 7.7) sec in IG (p < 0.0001) and 6.9 (SD 7.2) sec in the CG (p < 0.01). By contrast, there was a significant increase in endurance after the intervention for the 6MWT in both groups, with a 68.8 (SD 67.6) meters increase in the IG (p < 0.01) and a 48.1 (SD 48.6) meters increase (p < 0.05) for the CG group. However, no significant differences were evident between the groups (IG and CG) for any of these functional variables (p < 0.05).

**Table 3:** Functional outcomes following exoskeleton training or convectional training, and comparison among times and interventions

	Intervention Mean (SD)			Pairwise comparison (p-value, η2)	
	Baseline	Post-intervention	Change	Baseline vs. Post-intv	IG vs. CG
IG					
10MWT (m/s)	0.34 (0.16)	0.57 (0.41)	0.19 (0.16)	0.03*, η^2^ = 0.39	0.69, η^2^ = 0.04
TUG (s)	40.50 (19.70)	27.3 (15.57)	− 13.23 (7.71)	0.00*, η^2^ = 0.63	0.99, η^2^ = 0.16
6MWT (m)	114.67 (71.21)	183.56 (133.10)	68.79 (67.55)	0.00*, η^2^ = 0.43	0.75, η^2^ = 0.03
WISCI-II (0–20)	8.36 (3.98)	11.91 (4.25)	3.54 (2.65)	0.00*, η^2^ = 0.60	0.00*, η^2^ = 0.32
SCIM-III (0–100)	73.54 (7.76)	75.72 (10.05)	2.18 (3.37)	0.03*, η^2^ = 0.23	0.90, η^2^ = 0.01
CG					
10MWT (m/s)	0.41 (0.22)	0.52 (0.36)	0.12 (0.17)	0.04*, η^2^ = 0.21	
TUG (s)	37.37 (20.38)	30.42 (20.20)	− 6.9 (7.22)	0.01*, η^2^ = 0.33	
6MWT (m)	110.95 (72.16)	159.05(125.50)	48.10 (48.58)	0.02*, η^2^ = 0.27	
WISCI-II (0–20)	11.7 (3.8)	12.40 (4.45)	0.7 (1.49)	0.28, η^2^ = 0.05	
SCIM-III (0–100)	73.8 (5.49)	76.3 (6.10)	2.4 (2.7)	0.02*, η^2^ = 0.24	

CG: control group; IG: intervention group; *significant differences (p < 0.05). η^2^: calculated effect size. 10MWT: 10 Meters Walking Test; TUG: Test Up and Go; 6MWT: 6 Minutes Walking Test; WISCI-II: Walking Index Spinal Cord Injury-II; SCIM-III: Spinal Cord Independence Measurement-III

Significant differences in the WISCI-II were also observed for the “group” factor (F = 16.75, p < 0.001) and “group-time” interaction (F = 8.87; p = 0.01). Indeed, the post-hoc analysis revealed a significant increase of 3.5 (SD 2.65) points after intervention in the IG (p < 0.0001) but not in the CG when compared to the CG (0.7, SD 1.5 points; p = 0.285). WISCI-II details are shown in Table 4. Regarding the SCIM-III (Table 3), significant differences were found for the “time factor” (F = 11.6, p < 0.01) but not for the “group factor” (F = 0.36, p = 0.86) and for “time-group” interaction (F = 0.36, p = 0.87). Indeed, the post-hoc analysis revealed a significant increase in the global independence of 2.2 (SD 3.4) points in the IG (p < 0.05) and 2.4 (SD 2.7) points in CG (p < 0.05). However, no significant differences were found between the groups after the intervention (p = 0.90).

**Table 4:** WISCI II outcomes and technical aid used in functional gait test (10MWT, TUG, 6MinWT) at baseline and post-intervention time

Group	Participant	Baseline assessment				Post-intervention assessment			
		Use of devices	Braces	Physical assistance	WISCI II Score	Use of devices	Braces	Physical assistance	WISCI II Score
IG	1	Walker*	No	No	13	Two crutches*	No	No	16
	2	Parallel	Yes	One person	3	Parallel	No	No	4
	3	Walker	Yes	One person	6	Walker	No	No	13
	4	Two crutches	No	No	16	Two crutches	No	No	16
	5	Parallel	No	One person	4	Parallel	No	No	5
	6	Walker	Yes	One person	6	Walker	No	No	13
	7	Walker*	Yes	No	8	Two crutches*	No	No	16
	8	Two crutches*	Yes	No	12	One crutch*	Yes	Yes	15
	9	Walker	Yes	One person	6	Walker	Yes	One person	6
	10	Walker *	Yes	No	9	Two crutches*	Yes	No	12
	11	Walker*	Yes	No	9	Two crutches*	Yes	No	12
CG	1	Walker	Yes	One person	6	Walker	Yes	One person	6
	2	Walker	Yes	One person	6	Walker	Yes	One person	6
	3	Walker	Yes	No	9	Walker	Yes	No	9
	4	Two crutches	No	No	16	Two crutches	No	No	16
	5	Two crutches	No	One person	11	Two crutches	No	One person	11
	6	Two crutches	No	No	16	Two crutches	No	No	16
	7	Two crutches	Yes	No	12	Two crutches	No	No	16
	8	Two crutches*	No	No	16	One crutch*	No	No	19
	9	Walker	No	No	13	Walker	No	No	13
	10	Two crutches	Yes	No	12	Two crutches	Yes	No	12

CG: control group; IG: intervention group; *change of assistive device from pre- to post-intervention

10MWT: 10 Meters Walking Test; TUG: Test Up and Go; 6MWT: 6 Minutes Walking Test; WISCI-II: Walking Index Spinal Cord Injury- II

## Discussion

This article reports the results of a prospective, randomized, comparative clinical study, blinded to the evaluators conducted in a sample of individuals with incomplete SCI less than 1 year since injury to: (1) assess the safety and adherence to the robotic-assisted walking training as provided with of the device HANK exoskeleton by individuals with incomplete SCI; and (2) assess the changes in walking function after a training program with the robotic exoskeleton compared to traditional walking therapy. We hypothesized that the treatment with the exoskeleton would provide better functional walking outcomes than traditional overground gait therapy. To our knowledge, we present the first results of the effects on walking outcomes after training with robotic exoskeletons in SCI individuals less than 1 year since injury.

Although we estimated 24 individuals per group, we managed to recruit 23 and analyze 21 subjects. However, we present the available data, and our interpretation is unlikely to introduce bias because we did not conduct any interim analysis [45].

No serious adverse effects were found. Only a small number of skin issues were registered, and no participants withdrew from the study due to the use of the exoskeleton. All the clinical and functional variables assessed improved after treatment in both groups, although the WISCI-II improvement was greater in participants trained with the robotic exoskeleton. As it will be discussed below, this result has an important implication regarding the improvement mechanisms underlying the use of ambulatory robotic exoskeletons.

The HANK exoskeleton introduces some improvements with respect to the preceding Exo-H2 exoskeleton, the latter proved its feasibility to provide walking assistance in a sample of stroke individuals with preserved walking ability [31]. Furthermore, HANK exoskeleton shares most of its with other available exoskeletons e.g. active actuation of joint flexion/extension while restricting movement in the remaining degrees of freedom. Nevertheless, there are slight differences. Firstly, HANK is one of the few exoskeletons featuring active actuation at the ankle joint, although the potential effects on walking. Besides, small differences in structure design, strapping methods, actuator technology, sensors, and controller might influence the results. While there is still controversy regarding the clinical effectiveness of robotic-mediated rehabilitation interventions with respect to comparable, non-robotic interventions, our study, with all the similarities and dissimilarities, does neither aim to answer this big question nor test whether these small differences may cause different outcomes, but to add evidence on the impact of similar technology and treatment in a similar sample population, contributing to answering the fundamental question related to the outcomes of robotic walking rehabilitation conveyed with exoskeletons [28].

Here, we also demonstrate the feasibility of HANK to provide walking therapy in light of the changes in walking function in a sample of incomplete SCI individuals. The inclusion criteria focused on individuals with SCI less than 12 months since injury, not least because changes in the neurological situation can be expected and the effect of any therapy may be enhanced. In fact most of the patients were less than 6 months post injury. This supports the idea that the exoskeleton can be used to achieve better results in early gait rehabilitation. The optimal timing of treatment with exoskeletons, the duration and the protocols have yet to be defined. A recent study using an exoskeleton for gait training on acute SCI was yet focusing more on safety and feasibility than on neurological changes [19].

All of the participants except 2 completed the 15 training sessions, and these two withdrawals were not related to the intervention. In terms of the feasibility and adverse events, our results were similar to other studies [19, 27, 46, 47]. No swollen joints were observed during the training program, as occurred in previous studies [19, 27, 46] and only two participants in our cohort reported skin issues in contact areas that were rapidly resolved between the sessions. These data were similar to those reported with Ekso and Rewalk exoskeletons [19, 27, 47], yet better than those found elsewhere [46]. No falls occurred in our cohort, as seen in other experiences [19], however, some cases of pain in the neck and shoulder were reported as an eventual adverse event, probably more directly related to the musculoskeletal effort required when using technical aids for walking. None of the patients demanded a change on the external support, although all increased their ability of walking with the exoskeleton. Although in few cases it would have been possible to change the external support, we decided to maintain it to maximize the number of steps during the therapy: increasing the challenge due to the external support would had result in an increase of patient's fatigue and therefore a decrease on the number of steps. Besides, there is no clear evidence regarding when to transition from one external support to another (e.g., walker to two crutches) in the framework of exoskeleton walking therapy. Therefore, we can conclude that providing walking therapy the HANK exoskeleton is safe and feasible.

The overall pain and fatigue perceived here were recorded during robotic gait training through a VAS scale [19, 20]. A VAS is considered a valid measure to assess subjective perception and symptoms [35]. Self-perceived pain by participants during the sessions with HANK was rated lower than 3 cm on VAS scale (0–10 cm), which could therefore be considered as mild pain [35]. Fatigue was somewhat higher but still less than 4 cm and hence, it could be interpreted as moderate fatigue [35]. These results are consistent with other studies [18, 19, 27, 48] although several of them used Rate of Perceived Exertion on the Borg Scale.

As in previous studies [19, 20, 27, 46, 47, 49, 50] we found relevant improvements in LEMS and gait functional scales (10MWT, TUG and 6MWT) for the participants of both the IG and CG. As such, exoskeleton-driven walking therapy is apparently as effective than conventional walking treatment if these variables alone are considered. The only parameter that improved significantly in the IG was the WISCI-II. Although WISCI-II is considered to be less sensitive to changes in gait function than the 10MWT and TUG in incomplete SCI [51], significantly greater improvements in WISCI-II were produced in the IG than in the CG, suggesting an improvement in walking ability. It means that impairment in IG was lower than CG after training with exoskeleton because WISCI-II's levels reflect the severity of underlying impairment rather than the need for physical assistance, walking aids or braces [39]. It is well known the relationship between the severity of the impairment, reflected in the strength of leg muscles (LEMS) and the WISCI-II in subjects with SCI [37]. However no statistical significance was found for LEMS between both groups in our sample. So, this finding might suggest that training with the exoskeleton improves balance ability during walking. Given that robotic exoskeletons do not allow to maintain balance during walking, the use of external support devices is mandatory, which requires considerable effort to maintain balance during walking. Exoskeletons are indeed the only devices suitable for such intervention: passive orthoses require excessive energy, limiting the therapy time, and are prone to upper limb lesions. In our opinion, the main finding of our study is that the use of robotic exoskeletons for walking training indeed improves walking ability by promoting compensating and balance-related strategies, which can explain the WISCI-II improvement. Nevertheless, we did not measure other variables that could further explain this hypothesis—such as upper limb movement, balance, and/or EMG. Together, while participants in both the IG and CG group were able to walk faster (10MWT) and longer (6MWT), those in the IG needed less external assistance (WISCI-II). Nevertheless, the use of the exoskeleton has other disadvantages such as the time it takes to put it on, set the device and take it off, the high cost, the need for trained personnel, the batteries short life, etc. However, the cost–benefit analysis of this kind of technology and application is out of the scope of the article. We included neurological levels C2-L4 assuming that individuals with upper (UMN) and lower motor neuron (LMN) injury could be incorporated. It has been previously reported that patients with UMN and LMN can benefit from their gait training with robotics systems [52, 53] considering that functional improvements could not be exclusively accounted for by spinal circuitry responses to sensory input but rather than muscle strengthening play a fundamental role in individuals with incomplete SCI. Both aspects can be addressed by therapy with exoskeletons. Several questions remain still unanswered, such as what are the dose-time effects on walking ability, which criteria are best suited to design exoskeleton-driven walking therapies, and specifically, how and when to progress from parallel bars to a walker and then to crutches, or the impact of practicing advanced walking skills. One strength of this study is that we included participants with a specific condition (recent SCI, less than 1 year), trying to clarify the best indications for exoskeletons training.

### Study limitations

There were some limitations to the present study. Firstly, we did not set guidance force and step initiation detection specifically for each patient. Adapting robot guidance to the actual walking capacity of the patient, in other words to the patient’s movement as needed (Assist-as-Needed, AAN [54]), was proposed soon after robots for walking assistance were proposed, upon the Challenge Point Theory [54]. However, despite all the research effort made by the community, there is no evidence on how to adapt robot guidance to optimize the therapy [33]. Assessing the optimal level of assistance to provide during walking therapy requires measuring the actual walking capacity of the patient, which depends on several factors. Some of them can be estimated (e.g., joint force production from EMG readings), but some others are very difficult to estimate (e.g., impact of central fatigue or patient motivation on muscle force production). Some studies have proposed implementations of the AAN paradigm based on estimates of muscle fatigue [55], model-based estimates of lower limb biomechanics [56], or simply user-exoskeleton force interactions [57]. Nevertheless, contrary to inducing optimal assistance, implementations of the AAN paradigm have proven to induce "slacking" [58]. We still believe that a correct implementation of the AAN that provides optimal assistance to the patient's walking capacity would result in better therapeutic outcomes, but besides improving the estimates of the motor capacity and the effect of the impairment (muscle fatigue, complex and accurate neuromusculoskeletal models to estimate patient walking capacity, etc.), other factors should be also accounted for, and introduced into the calculation of the guidance (central fatigue, motivation, biomechanical and neural complexity, etc.). In this sense, next coming research will investigate how to optimize the device to actual patient functional requirements [58].

The sample size was not adequate since we estimated 24. Besides, no stratification based on the time since the injury or the level of injury could be made. The IG appears to be younger with a shorter injury duration, which could potentially favor more change. However, differences for demographic variables did not reach statistical significance. This could also be attributable to the limited study size. Another limitation, no follow-up evaluation after 6 months was registered. Furthermore, the recruitment period and the number of training sessions were limited due to logistics and time constraints. Likewise, it was not possible to carry out a multi-center study as the exoskeleton is not available at other centers. Finally, we also acknowledge that the intervention could not be blinded to the patient, therefore risking a placebo effect.

## Conclusion

The results of a prospective, randomized, comparative study of walking training with the lower limb robotic ambulatory exoskeleton HANK, are presented. This study provides evidence of its safety and feasibility for gait training in patients with SCI less than 1 year since injury. We found that both the IG and CG subjects were able to walk faster and longer after the training programs, yet the IG patients needed less external assistance. In the light of these data, the mechanisms responsible for the improvements in exoskeleton-driven interventions should be confirmed in future multi-center efficacy trials with larger sample sizes.

## Data Availability

The dataset analyzed during the current study are available from the corresponding author on reasonable request.

## Abbreviations

6MWT: 6-Minute Walk Test
10MWT: 10-Meter Walk Test
AIS: American Spinal Cord Injury Association Impairment
CG: Control group
IG: Intervention group
ISNCSCI: International Standards for Neurological Classification of Spinal Cord Injury
LEMS: Lower extremity motor score
SCI: Spinal cord injury
SCIM-III: Spinal Cord Independence Measure (second version)
TUG: Timed Up and Go
VAS: Visual Analogue Scale
WISCI-II: Walking Index for Spinal Cord Injury (second version)
