# Correction: Exoskeleton-based training improves walking independence in incomplete spinal cord injury patients: results from a randomized controlled trial

**DOI:** 10.1186/s12984-023-01281-x

**Published:** 2023-11-18

**Authors:** Ángel Gil-Agudo, Álvaro Megía-García, José Luis Pons, Isabel Sinovas-Alonso, Natalia Comino-Suárez, Vicente Lozano-Berrio, Antonio J. del-Ama

**Affiliations:** 1 Biomechanics and Technical Aids Department, National Hospital for Paraplegics, SESCAM, Finca la Peraleda s/n, 45071 Toledo, Spain; 2 Physical Medicine and Rehabilitation Department, National Hospital for Paraplegics, SESCAM, Toledo, Spain; 3 Neurorehabilitation and Biomechanics Unit (HNP‑SESCAM), Associate Unit CSIC, Toledo, Spain; 4 Toledo Physiotherapy Research Group (GIFTO), Faculty of Physiotherapy and Nursing, Castilla La Mancha University, Toledo, Spain; 5 Legs and Walking Lab, Shirley Ryan Ability Laboratory (Formerly Rehabilitation Institute of Chicago), Chicago, IL USA; 6 Department of Physical Medicine and Rehabilitation, Feinberg School of Medicine, Northwestern University, Chicago, IL USA; 7 Department of Biomedical Engineering, McCormick School of Engineering and Applied Science, Northwestern University, Chicago, IL USA; 8 Department of Mechanical Engineering, McCormick School of Engineering and Applied Science, Northwestern University, Chicago, IL USA; 9 Neural Rehabilitation Group, Cajal Institute, Spanish National Research Council (CSIC), Madrid, Spain; 10 Electronic Technology Area, Rey Juan Carlos University, Mostoles, Spain

**Correction: Journal of NeuroEngineering and Rehabilitation (2023) 20:36** **https://doi.org/10.1186/s12984-023-01158-z**

In the original version of this article [1], WISCI-II variable values in the “Baseline and Post-intervention” row of Table 3 has been updated and same has been shown below.

**Table 3:** Functional outcomes following exoskeleton training or convectional training, and comparison among times and interventions

		Intervention Mean (SD)			Pairwise comparison (p-value, η2)	
		Baseline	Post-intervention	Change	Baseline vs. Post-intv.	IG vs. CG
IG	10MWT (m/s)	0.34 (0.16)	0.57 (0.41)	0.19 (0.16)	0.03*, η2 = 0.39	0.69, η2 = 0.04
	TUG (s)	40.50 (19.70)	27.3 (15.57)	− 13.23 (7.71)	0.00*, η2 = 0.63	0.99, η2 = 0.16
	6MWT (m)	114.67 (71.21)	183.56 (133.10)	68.79 (67.55)	0.00*, η2 = 0.43	0.75, η2 = 0.03
	WISCI-II (0–20)	8.36 (3.98)	11.91 (4.25)	3.54 (2.65)	0.00*, η2 = 0.60	0.00*, η2 = 0.32
	SCIM-III (0–100)	73.54 (7.76)	75.72 (10.05)	2.18 (3.37)	0.03*, η2 = 0.23	0.90, η2 = 0.01
CG	10MWT (m/s)	0.41 (0.22)	0.52 (0.36)	0.12 (0.17)	0.04*, η2 = 0.21	
	TUG (s)	37.37 (20.38)	30.42 (20.20)	− 6.9 (7.22)	0.01*, η2 = 0.33	
	6MWT (m)	110.95 (72.16)	159.05 (125.50)	48.10 (48.58)	0.02*, η2 = 0.27	
	WISCI-II (0–20)	11.7 (3.8)	12.40 (4.45)	0,7 (1.49)	0.28, η2 = 0.05	
	SCIM-III (0–100)	73.8 (5.49)	76.3 (6.10)	2.4 (2.7)	0.02*, η2 = 0.24	

CG: control group; IG: intervention group; *significant differences (p < 0.05). η2: calculated effect size; 10MWT: 10 Meters Walking Test; TUG: Test Up and Go; 6MWT: 6 Minutes Walking Test; WISCI-II: Walking Index Spinal Cord Injury-II; SCIM-III: Spinal Cord Independence Measurement-III

The original article has been corrected.
